# Two themes in thyroid cancer: artful diagnosis and shortened lives

**DOI:** 10.1590/2359-3997000000275

**Published:** 2022-05-01

**Authors:** Cristiane Gomes Lima, Leonard Wartofsky

**Affiliations:** 1 MedStar Health Research Institute Georgetown University School of Medicine Washington DC USA MedStar Washington Hospital Center and MedStar Health Research Institute, Georgetown University School of Medicine, Washington, DC, USA

Patients presenting with a thyroid nodule are common in the clinical practice of endocrinologists, even for those who are not thyroidologists. When seeing a patient with a thyroid nodule, the question that typically occurs first is whether the nodule could be malignant and how can the diagnosis be most efficiently and accurately determined. Then, once a diagnosis of cancer might be confirmed, and discussion turns to details about management, the next prominent question in the patient’s mind relates to their prognosis. The importance of these two questions, precise diagnosis and prognosis, forms the basis for two papers appearing in this issue of the *Archives of Endocrinology and Metabolism*.

Detection of thyroid nodules has been increasing significantly due to the more widespread use of ultrasonography of the neck. Our professional society guidelines recommend fine-needle aspiration (FNA) as the procedure of choice for nodules > 1 cm, and the routine use of thyroid ultrasonography to characterize the nodules. Yet the subjective nature of sonogram evaluations and the lack of uniformity in the reports of the characteristics of thyroid nodules may be troublesome for management decision-making. This is the setting in which Delfim and cols. (1) propose and offer a new classification system to distinguish the ultrasound features between benign and malignant thyroid nodules. In fact, similar efforts to develop a thyroid imaging reporting and data system (TIRADS) to categorize thyroid nodules and evaluate their risk of malignancy date back to 2009 (2-6), in parallel to what has been done for breast imaging with the development of the Breast Imaging Reporting and Data System (BI-RADS) (7). The Korean Society of Thyroid Radiology has recently revised its recommendations, the K-TIRADS (8) and the American College of Radiology has just released a white paper of the TI-RADS Committee (9). The current guidelines of the American Thyroid Association (ATA) (10) recommend the use of sonographic patterns, instead of isolated sonographic features, to estimate the risk of malignancy of thyroid nodules.

Each of the latter reports strive to present a standardized system for analyzing and reporting thyroid ultrasound that could result in greater diagnostic specificity. Strengths and weaknesses of the various systems relate to whether the studies are either prospective or retrospective, the readings are by single or multiple radiologist investigators, the use of different techniques for the evaluation of the nodules and varying statistical models, and correlation to different categories of the Bethesda system. Regardless of which system might be ultimately adopted, the need for a standardized terminology is rational and functional. A committee of the American College of Radiology, composed by radiologists with expertise in thyroid imaging, has developed a descriptive lexicon of the sonographic characteristics of thyroid nodules (11). The Korean Society of Thyroid Radiology has also recommended terminology and defined sonographic features of nodules in its revised consensus (8). In their paper, Delfim and cols. use a well defined terminology to describe their scoring system for ultrasound features that led to their proposed TI-RADS system, based on statistical analysis and a weight conception process. In this process, certain features of the nodules, such as hypoechogenicity and microcalcifications, received a higher score than central vascularization, reinforcing the relevance of B-mode ultrasound over Doppler mode characteristics. The exclusion of indeterminate nodules from their analysis is a relative weakness of their study. However, it is generally acknowledged that imaging reporting systems are not supposed to be superior to the cytological evaluation of a thyroid nodule.

Any TIRADS system should remain flexible and learn a lesson from its “older brother”, the BI-RADS system (7,12-15), insofar as being a “living” document, founded on logical and evidence-based data but open to updates as new data are acquired (16).

The second paper in this issue, by Leite and cols. (17), analyzes deaths related to differentiated thyroid cancer (DTC). Current trends in management have moved us to be less aggressive in the treatment and management of DTC, practicing the so called “less is more” philosophy. However, as the authors discuss, the extremely low mortality of this type of cancer “is balanced by its high prevalence, so the number of deaths cannot be overlooked”. Indeed, endocrinologists who work in referral services of thyroid cancer and see many high risk patients appreciate the mortality risk. It is remarkable that the series of Leite and cols. included more patients with follicular cancer (with associated risk of distant metastases) than is commonly seen. Also noteworthy is the fact that 4 out of 33 patients had stage T1 disease, while 2 out of 33 patients had stage T2, i.e., low or intermediate risk patients that would be considered (by the current “less is more” philosophy) for less aggressive treatment (*e.g*., lobectomy instead of total thyroidectomy, and no radioiodine ablation), in keeping with the new ATA guidelines (10). Clearly, all T1 patients do not behave the same. Those low risk patients with ultimate poor outcomes could be detected by periodic risk assessment in order to detect those patients initially stratified as low risk who may develop an unexpected aggressive course of the disease.

The light at end of the tunnel may derive from a beacon of promise from molecular diagnosis, either for the management of nodules or for the follow-up of proven cancer patients at either low or high risk. When this diagnostic tool becomes more refined and more accessible, it will possibly identify the most suspicious nodules and those cancer patients who need a more aggressive treatment approach during follow-up. A refined and comprehensive molecular analytic approach to the thyroid nodule will provide true precision medicine for our patients and the best hope for maximizing benefit, reducing risk, and achieving good outcomes.

To echo Goethe, dealing with our patients’ fear of cancer when a nodule is discovered calls forth the art of the true physician, especially for those patients whose life ultimately will be foreshortened after presentation with metastases, for we have only imprecise information on which to act and affect their outcome, and only a limited time to do so [Von Goethe, J. “Art is long, life short, judgment difficult, occasion transient.” (18)].
